# Somatic frameshift mutation in *PIK3CA* causes CLOVES syndrome by provoking PI3K/AKT/mTOR pathway

**DOI:** 10.1186/s41065-021-00184-y

**Published:** 2021-06-01

**Authors:** Wei Yan, Bin Zhang, Huijun Wang, Ran Mo, Xingyuan Jiang, Wen Qin, Lin Ma, Zhimiao Lin

**Affiliations:** 1 Department of Dermatology, Peking University First Hospital, Beijing Key Laboratory of Molecular Diagnosis on Dermatoses and National Clinical Research Center for Skin and Immune Diseases, 8 Xishiku St, Beijing, 100034 China; 2 Department of Dermatology, Beijing Children’s Hospital, Capital Medical University, National Center for Children’s Health, No.56 Nanlishi Road, Xicheng District, Beijing, 100045 China; 3 Department of Dermatology, Zhengzhou University, Affiliated Children’s Hospital, Henan Children’s Hospital, Zhengzhou Children’s Hospital, Zhengzhou, 450000 Henan China

**Keywords:** PIK3CA, CLOVES syndrome, Somatic mutation, PI3K/AKT/mTOR pathway

## Abstract

**Background:**

CLOVES syndrome (OMIM# 612918) is a rare overgrowth disorder resulted from mosaic gain-of-function mutations in the *PIK3CA* gene. All the reported CLOVES-associated *PIK3CA* mutations are missense mutations affecting certain residues. We aim to investigate underlying mutation and its pathogenicity in a patient with CLOVES syndrome and to evaluate the inhibitory effects of the PI3K/AKT/mTOR pathway inhibitors.

**Results:**

We performed whole-exome sequencing (WES) and Sanger sequencing to detect underlying somatic mutations in the skin lesion of the patient. Quantitative real-time PCR (qRT-PCR) was employed to evaluate the mRNA abundance of *PIK3CA* in the patient’s skin lesion. AKT phosphorylation level assessed by immunoblotting of lysates from transiently transfected cells was performed to evaluate the *PIK3CA* mutations and inhibitory effects of PI3K/AKT/mTOR pathway inhibitors. A somatic frameshift mutation c.3206_3207insG (p.X1069Trpfs*4) in *PIK3CA* was identified in the genomic DNA extracted from the vascular malformation sample of the patient. This mutation affects the canonical stop codon of *PIK3CA* (NM_006218.4) and is predicted to produce a prolonged protein with four additional residues. qRT-PCR demonstrated that the mRNA expression levels of the patient’s affected skin tissue were comparable compared to the normal control. In vitro studies revealed that p.X1069Trpfs*4 mutant exhibited increased AKT phosphorylation significantly to that of the wildtype, which could be inhibited by PI3K/AKT/mTOR pathway inhibitors.

**Conclusions:**

We have identified the first frameshift mutation in *PIK3CA* that causes CLOVES syndrome, which was confirmed to overactive PI3K/AKT/mTOR pathway by transient transfection assays. We also provided more evidence of ARQ092 to be a potential therapeutic option for PROS in vitro.

## Background

Phosphatidylinositol 3‑kinases (PI3Ks) are a family of lipid kinases involved in the regulation of multiple cellular processes such as cell proliferation, differentiation, apoptosis, motility, and metabolism. It can be divided into three classes (Class I, II, III), and Class I can be further subdivided into Class IA and Class IB. The Class IA PI3K is a heterodimer composed of a p110 catalytic subunit and a p85 regulatory subunit. With the activating signals of receptor tyrosine kinase (RTK) or G‑protein coupled receptor (GPCR), p85 relieve the inhibition of p110 and Class IA PI3Ks are recruited to the plasma membrane. The phosphorylate phosphatidylinositide (PtdIns) 4,5‑bisphosphate (PIP2) is generated to PtdIns(3,4,5)P3 (PIP3) as a result of p110 subunit activation. PIP3 acted as a second messenger, which leads to the phosphorylation of AKT at residue Thr308 and Ser473 respectively. The mammalian target of rapamycin (mTOR), as a conserved serine/threonine kinase, which composed of two protein complexes, mTORC1 and mTORC2. mTORC1 can be activated directly by phosphorylated AKT at Ser2448 while mTORC2 has been proved to phosphorylate AKT at residue Ser473 via a feedback loop. For fully AKT activation, the phosphorylation status of AKT (Ser473 and Thr308) is necessary, which leads to the overactivation of the PI3K/AKT/mTOR pathway [1–3].

Somatic mutations in *PIK3CA*, the gene encoding the p110α, give rise to the abnormal PI3K/AKT/mTOR pathway activation [4–6]. Such mutations have been identified in some cancers and overgrowth disorders termed PIK3CA-related overgrowth spectrum (PROS) [7]. PROS comprises a group of disorders characterized by heterogeneous segmental overgrowth phenotypes with or without vascular anomalies due to somatic *PIK3CA* activating mutations. CLOVES syndrome, one of the PROS, is characterized by congenital lipomatous overgrowth (CLO), vascular malformation (V), epidermal nevi (E), and scoliosis/spinal malformation (S) [8–12]. Mosaic mutations in hot spot codons of *PIK3CA* (542, 545, 1047) account for the majority of the individuals with CLOVES syndrome [13]. Given that the hyperactivation of the PI3K/AKT/mTOR pathway is an important leading cause of numerous solid tumors and overgrowth syndromes, targeting this pathway signaling is an effective therapy for these disorders. BYL719 (Alpelisib), which works as a selective PI3Kα inhibitor, is a treatment for *PIK3CA*-associated cancers [14, 15]. After treatment with BYL719 in a clinical trial, all nineteen patients with PROS demonstrated smaller vascular malformations, reduced hypertrophy or other improvements, and a low rate of substantial side effects were observed, which first confirmed BYL719 is clinically effective and safe as a potential therapeutic strategy for PROS [16]. ARQ092 is a highly selective allosteric AKT inhibitor that has been evaluated in clinical trials for several PI3K/AKT driven tumors and overgrowth syndromes. Previous studies showed that ARQ092 attenuates AKT activation by disrupting membrane translocation of inactive AKT form and leading to dephosphorylation of active form [17]. As an allosteric mTOR inhibitor, rapamycin plays an important role in regulating AKT phosphorylation by inhibiting the function of mTORC1 predominantly [18, 19].

Herein, we report a patient with CLOVES syndrome with a somatic mutation (c.3206_3207insG, p.X1069Trpfs*4) in *PIK3CA*. Interestingly, this is a frameshift mutation that affects the canonical stop codon and produces a prolonged protein with four additional residues. By performing quantitative real-time PCR and western blot analysis, we demonstrated that there was no mRNA decay of *PIK3CA* in the patient’s affected skin lesion, while an obvious elevation in AKT phosphorylation was determined, suggesting a gain-of-function property of the mutation.

## Materials and methods

### Ethical approval and subjects

This study was approved by the Clinical Research Ethics Committee of Peking University First Hospital. The patient was recruited from our clinic and written informed consent was obtained from her guardians in adherence to the Declaration of Helsinki Principles.

### Mutational analysis and quantitative real-time PCR

The peripheral blood sample and biopsy samples of affected skin tissues from the patient were collected. Skin lesions were separated into epidermis and dermis according to a standard method. We performed whole-exome sequencing (WES) using genomic DNA extracted from the dermis with vascular malformation lesions. The criteria for the selection of candidate variants was as follows: (a) total read depth across the position ≥ 20, mutant reads ≥ 5, mutant reads frequency ≥ 1%. (b) nonsynonymous variants; (c) variants absent or with a minor allele frequency < 1% in any of the public databases (the [1000G], [ExAC], [ESP 2500 and 6500] and the [gnomAD]); (d) variants predicted to be “damaging”, “probably damaging” or “disease-causing” by at least one in silico tools, including SIFT, Mutation Taster, PolyPhen and GERP. Sanger sequencing was used to confirm the candidate pathogenic mutation found by exome sequencing.

Total RNA from the individual’s dermis of the affected skin samples was extracted using TRIzol reagent (Invitrogen) and equal amounts of RNA from each sample were reverse transcribed to cDNA according to the manufacturer’s instructions. To access the consequence of the mutation, we performed quantitative real-time PCR analysis of *PIK3CA* using the cDNA samples. Amplicons were quantified with Power SYBR Green PCR Master Mix (ABI) using a CFX Connect Real-Time System (Bio-Rad), and expression levels were normalized to those of GAPDH.

### Cell culture

HEK293 cells were purchased from ATCC (American Type Culture Collection, USA) and incubated in Dulbecco modified Eagle medium supplemented with 10% fetal bovine serum containing 100 U/ml of penicillin and 100 U/ml of streptomycin at 37 °C in an atmosphere of 5% CO2.

### Plasmids constructions and transfection

For exogenous expression in mammalian cells, human *PIK3CA* cDNA was cloned into pLenti-CMV vector encoding a C-terminal 3 × Flag tag. The point mutations in *PIK3CA* (c.501G > C, c.1624G > A, c.3140A > G, and c.3206_3207insG [Arg115Pro, Glu542Lys, His1047Arg, and X1069Trpfs*4]) were generated following the manufacturer’s protocol of C214 Mut Express® II Fast Mutagenesis Kit V2. HEK293 cells were seeded into six-well plates at a density of 4 × 105 cells per ml. Transfections with 3 μg of plasmids of empty vector, PIK3CA-wildtype (PIK3CA-WT), PIK3CA-Arg115Pro, PIK3CA- Glu542Lys, PIK3CA- His1047Arg, and PIK3CA-X1069Trpfs*4 were performed in the six-well plates when cell confluence reached 80%.

### Western blot analysis

Forty-eight hours after transfection, cells were washed twice in PBS and lysed in lysis buffer. Total proteins were extracted from the cultured cells and the protein concentration was measured using the Bradford method according to the manufacturer’s recommendations. All the protein samples were heated with loading sample buffer and reducing agent buffer( NuPAGE) for 10 min at 70 °C. The nitrocellulose filter (NC) membranes transferred with the proteins were blocked with 5% skim milk in TBST for 1 h at room temperature and then incubated with first antibodies overnight at 4 °C. Specific first antibodies: Mouse Anti-FLAG monoclonal antibody(Sigma; F3165; 1:1000), phospho-AKT Ser473 Rabbit monoclonal antibody(CST; #4060; 1:2000), phospho-AKT Thr308 Rabbit monoclonal antibody(CST;#13,038; 1:1000), Rabbit Anti-AKT monoclonal antibody (CST; #4685; 1:1000), Rabbit Anti-ERK monoclonal antibody (CST; #4695;1:1000), phospho-ERK Thr202/204 Rabbit monoclonal antibody (CST; #4370; 1:2000), Rabbit Anti-STAT1 monoclonal antibody (CST; #9176; 1:1000), phosphor-STAT1 Tyr701 Rabbit monoclonal antibody (CST; #9167; 1:1000). Second antibodies: horseradish peroxidase (HRP)-linked anti-mouse/rabbit antibody(ZB-2305/ZB-2301; 1:5000). Mouse Anti-GAPDH monoclonal antibody (TA-08; 1:1000) was used as a control.

### Inhibitors treatment

We also tested the inhibitory effects of three inhibitors (BYL719, ARQ092, rapamycin) related to the PI3K/AKT signaling pathway. HEK293 cells were seeded into six-well plates and then transfected following the standard method. Then cells were then treated with full media containing different concentrations of three inhibitors respectively: 10 μM BYL719, 5 μM ARQ092, 15 μM rapamycin approximately 48h after transfection. Protein samples were collected from cell lysates 6 or 12h after treatment with inhibitors and Western blot analysis was also performed to assess inhibitory effects.

## Results

### Clinical features and therapeutic approaches

**Fig. 1:**
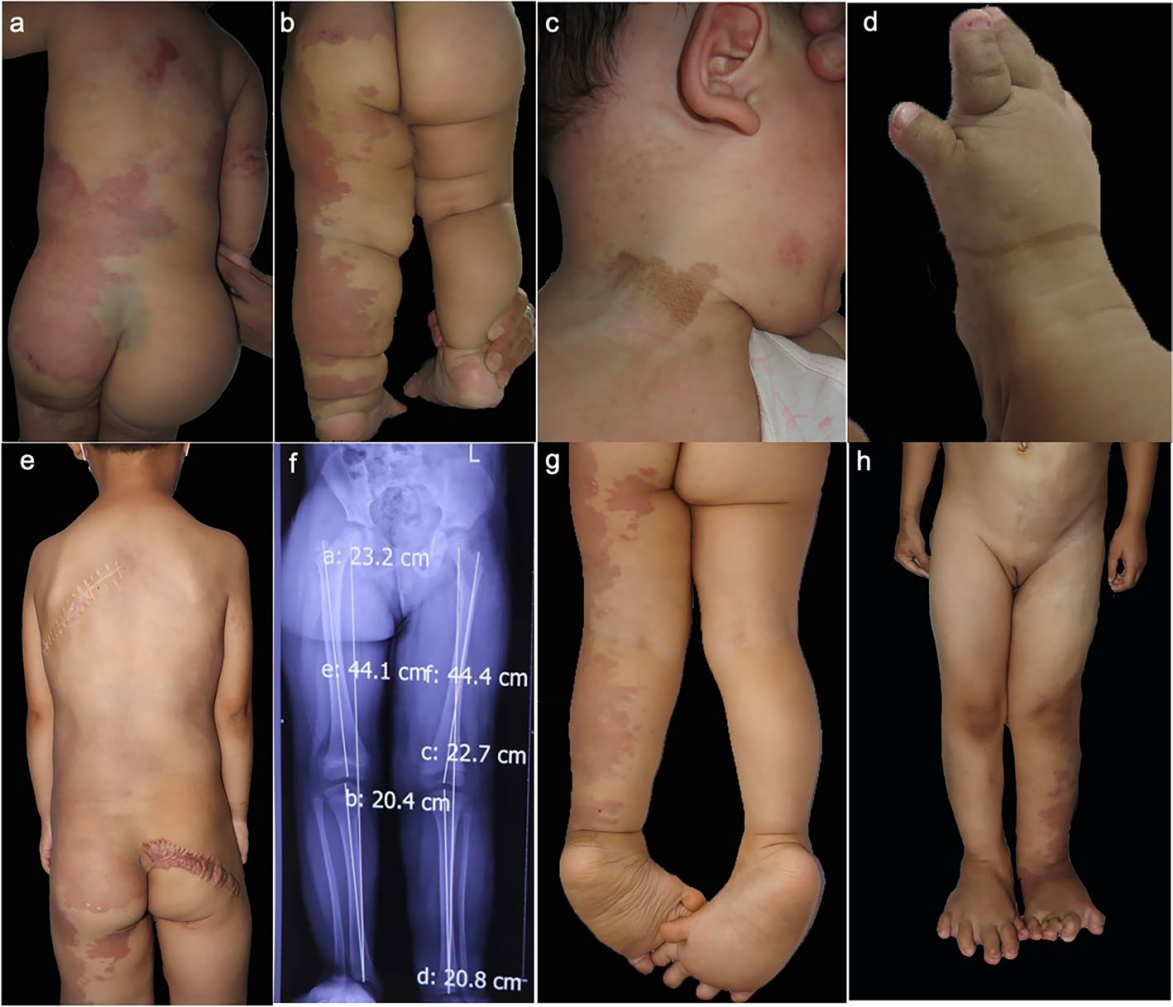
Clinical features of the patient. **a**-**b** The patient showed irregularly extended capillary malformations and asymmetrical hypertrophy of the lower limb. **c** Epidermal nevi. **d** Sandal gap toe and wide foot with second and third toes macrodactyly caused by skeletal elongation and adipose overgrowth. **e** Scoliosis and surgical removal of lipomatous masses. **f** X-ray examinations confirmed the asymmetrical overgrowth. **g** Decreased vascular anomalies and fat overgrowth of her lower limb after oral rapamycin treatment. **h** Removal of the left third toe by surgical amputation

The patient was a 7-year-old girl diagnosed with CLOVES syndrome based on the typical clinical characteristics and genetic testing results. Her family history was unremarkable. She was born by spontaneous vaginal delivery at 37 weeks of gestation. At birth, she presented with a capillary malformation on the left side of the body, her lower limb, and buttock (Fig. 1a). Other manifestations include congenital lipomatous overgrowth (right buttock), vascular malformation mass (left side of the back), and epidermal nevi (neck) (Fig. 1b-c). Scoliosis and broad feet with widened first interdigital space were observed (Fig. 1d-e). Surgical reduction of vascular malformation on her left back was performed at around 4 years old because of developed compression symptoms. Since then the patient had undergone multiple surgeries for removal of the lipomatous mass in the gluteal area (Fig. 1d-e). The X-ray radiology examination revealed evident hypertrophy of the left leg and lymphatic malformations extending into the retroperitoneum (Fig. 1f). Symptoms of claudication caused by the unilateral deformation worsened with age.

With informed consent, the patient was treated with oral sirolimus (rapamycin) at 1.5 mg/m^2^ per day, administered every 12 h at a serum concentration of 7–15 ng/ml. After 1 year of treatment, a significant decrease in the vascular malformation area of her lower limb could be seen (Fig. 1g). No obvious adverse effect was observed during her treatment with sirolimus during our following visit. Meanwhile, the patient received surgical amputation of the left third toe, which improved her physical mobility and life quality (Fig. 1h)

### A frameshift *PIK3CA* mutation escaping nonsense-mediated mRNA decay

**Fig. 2:**
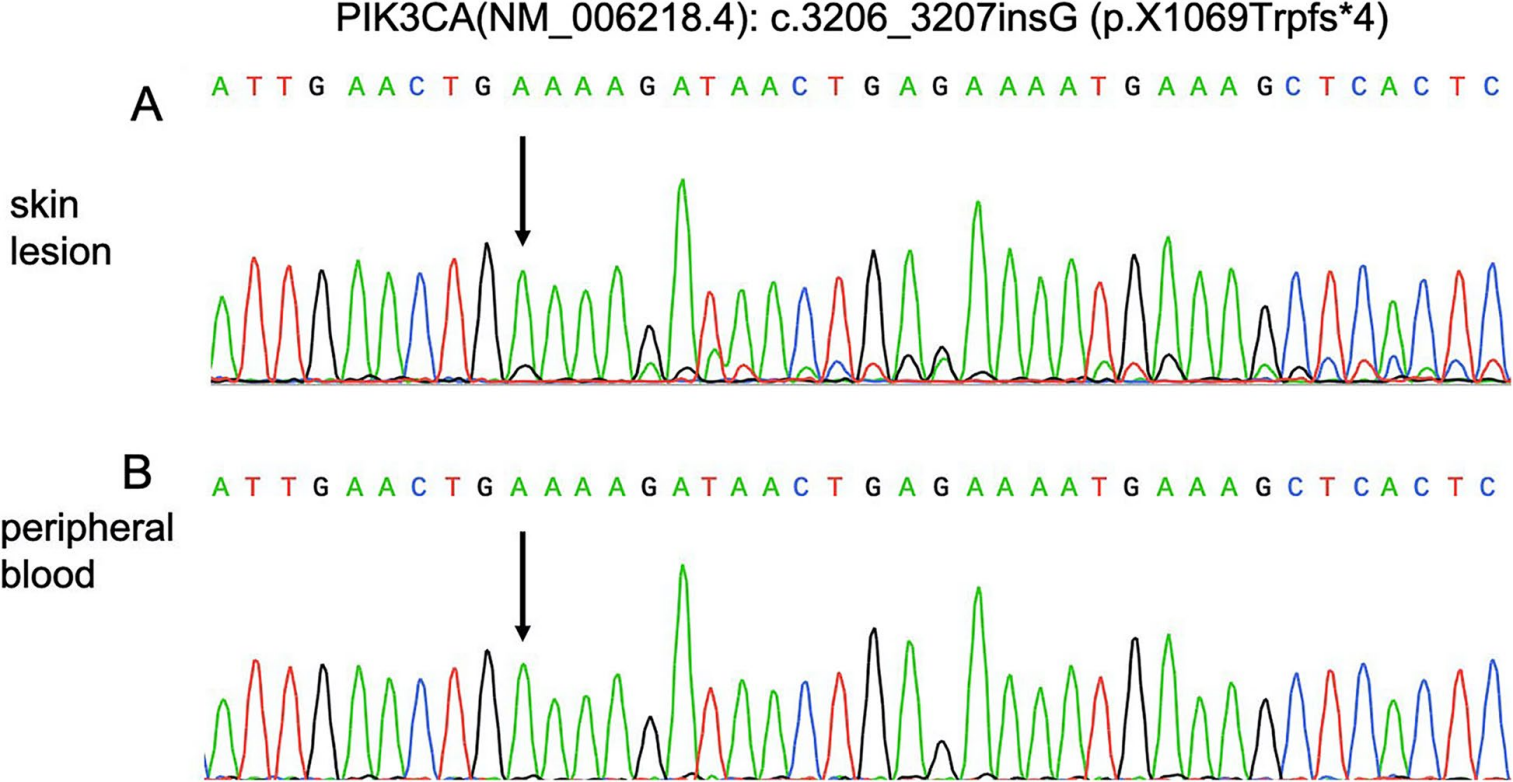
Sequencing results of the patient. **a** Mosaic *PIK3CA* mutation of c.3206_3207insG (p.X1069Trpfs*4) detected in the genomic DNA of the patient’s skin lesion sample. **b** This mutation was absent in the genomic DNA from peripheral blood

**Fig. 3:**
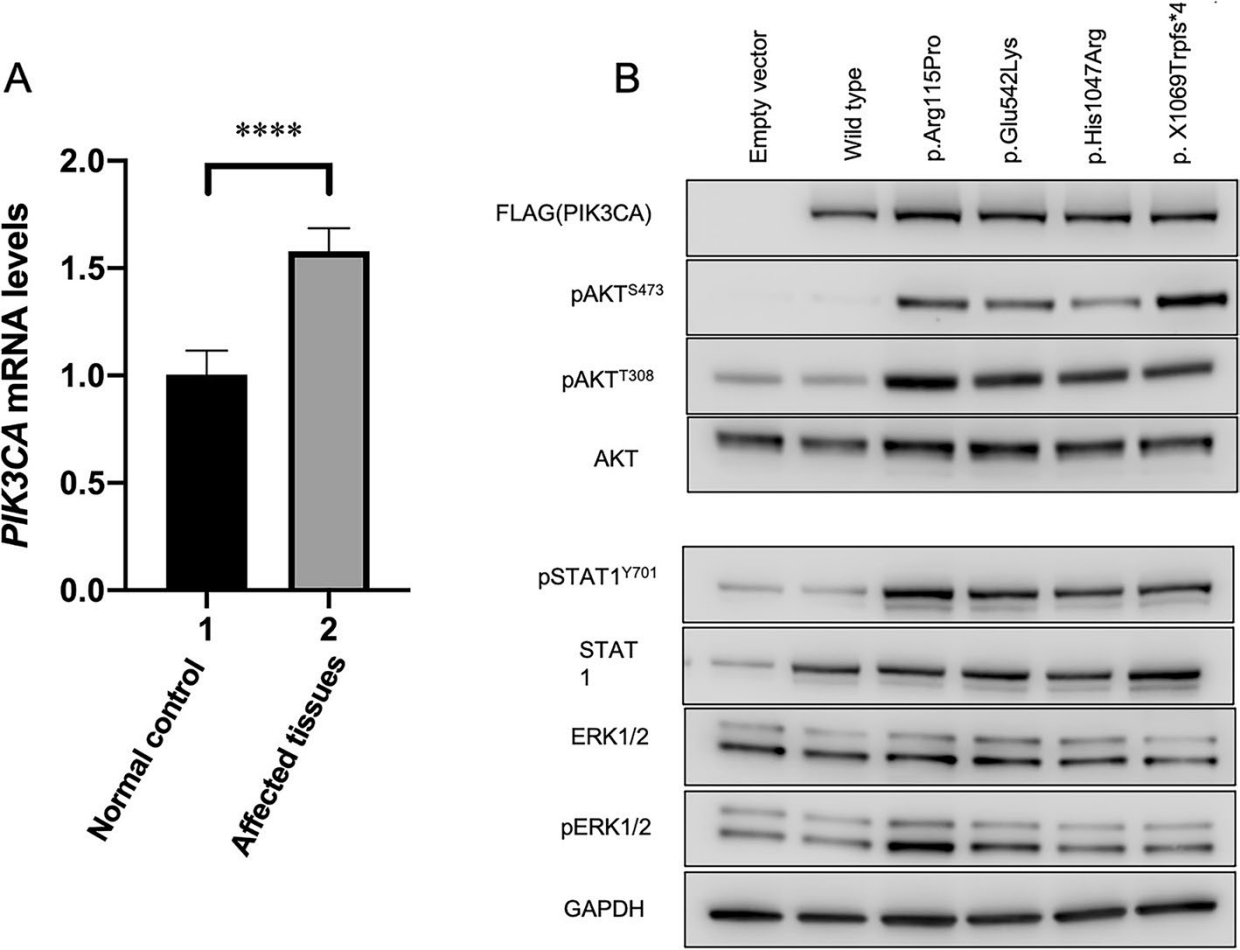
qRT-PCR results and representative western blots of PI3K/AKT/mTOR pathway. **a** The expression of *PIK3CA* mRNA in the patient’s lesion of dermis was comparable to that of an age and sex-matched control. **b** The GAPDH was used as a control. The phosphorylation of AKT at Ser473 and Thr308 is significantly elevated in the four mutations compared with the empty vector and wild-type (WT)

A frameshift mutation c.3206_3207insG (p.X1069Trpfs*4) in *PIK3CA* was detected in the patient’s tissue sample via WES and the mutant allele frequency was estimated as approximately 20%, which was confirmed by Sanger sequencing (Fig. 2a). This mutation was not present in the blood sample of the patient (Fig. 2b). The expression of *PIK3CA* mRNA in the patient’s lesion was comparable to that of an age and sex-matched control, as demonstrated by our qRT-PCR results (Fig. 3a). This insertion frameshift mutation affected the stop codon “TGA” of *PIK3CA* and our result suggested that the mutation escaped nonsense-mediated mRNA decay.

### *PIK3CA* frameshift mutation results in elevated AKT phosphorylation

Compared to the empty vector and PIK3CA-WT, the expression levels of p-AKT^T308^ and p-AKT^S473^ were both significantly elevated in the four *PIK3CA* variants (PIK3CA-Arg115Pro, PIK3CA- Glu542Lys, PIK3CA- His1047Arg, and PIK3CA-X1069Trpfs*4). Previous studies indicate that the PI3K/AKT/mTOR pathway interacts with multiple signaling pathways involved in cell proliferation and differentiation including ERK/MAPK and JAK/STAT. Thus, our Western blot analysis also accessed the phosphorylation of ERK and STAT. Compared to PIK3CA-WT, *PIK3CA* variants expressed higher levels of pSTAT1 while there is no obvious difference in pERK1/2 expression levels (Fig. 3b). STAT1, as the first member of STAT family, plays an oncogenic role in patients with breast and ovarian cancers [20]. Previous reports revealed that patients with higher expression levels of STAT1 or phospho- STAT1 have a worse outcome compared with patients with lower expression levels of those [21, 22]. Under these results, we confirmed that this frameshift mutation c.3206_3207insG (p.X1069Trpfs*4) could lead to the overactivation of the PI3K/AKT/mTOR pathway as a result of elevated AKT phosphorylation as well as reported hot spot mutations. Moreover, we concluded that this mutation led to a slight overactivation effect on JAK/STAT signaling pathway.

### AKT phosphorylation inhibited by BYL719, AR092, and rapamycin

**Fig. 4:**
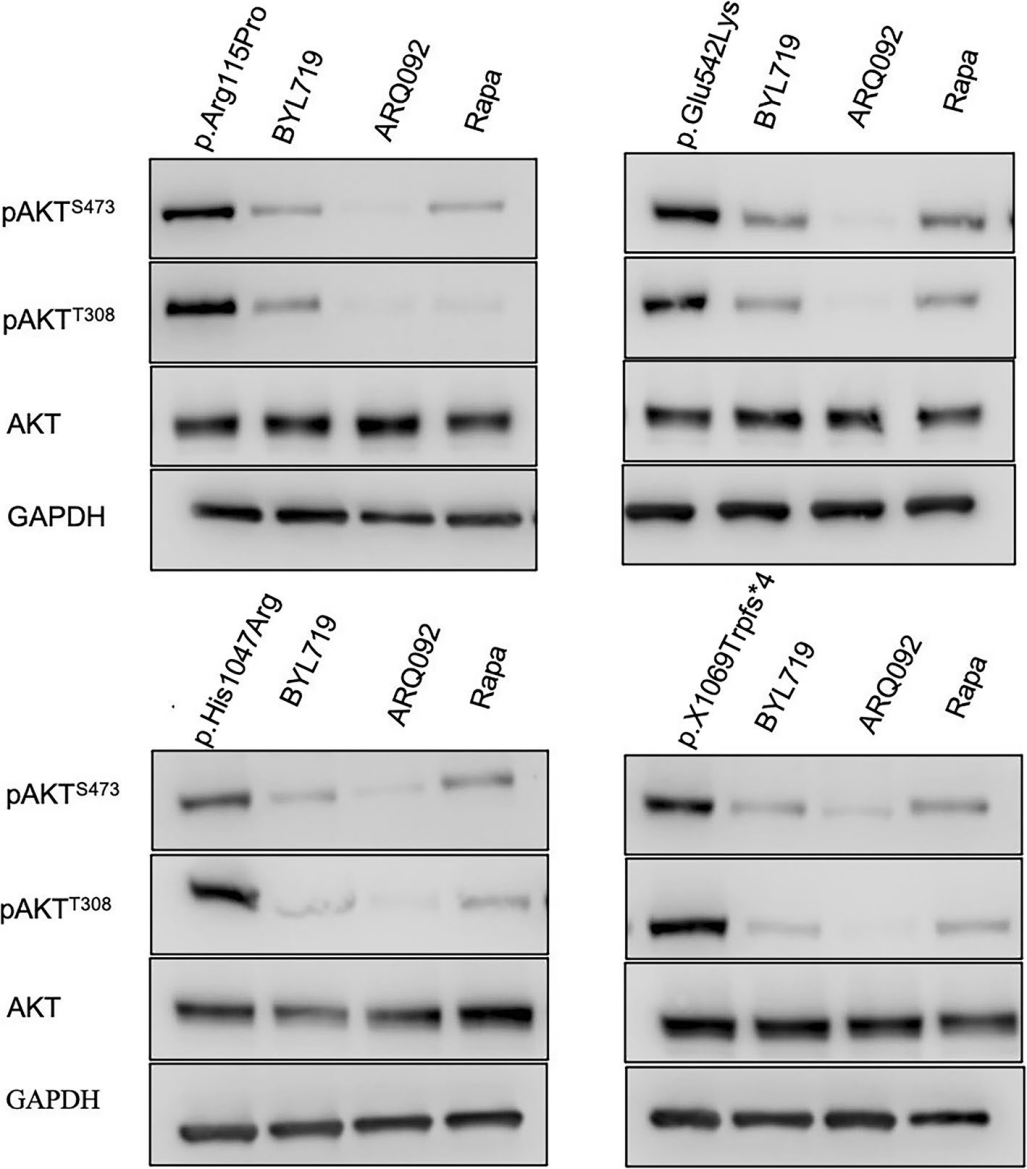
Comparison of AKT activity inhibition by three inhibitors. All three inhibitors showed potency to decrease the phosphorylation of AKT while ARQ092 has a stronger inhibitory effect than BYL719 or rapamycin

The expression levels of p-AKT^T308^ and p-AKT^S473^ were both significantly decreased after treated with any of the three compounds BYL719, ARQ092, and rapamycin. Compared with BYL719 or rapamycin, a lower dose of ARQ092 exhibited stronger inhibitory effects on decreasing phosphorylation of AKT at Thr308 and Ser473 (Fig. 4). Our results suggested that ARQ092 could be considered as a potential medication measurement for CLOVES syndrome or other disorders caused by hyperactivation of the PI3K/AKT/mTOR pathway.

## Discussion

In this study, we reported a patient with CLOVES syndrome caused by a somatic frameshift mutation c.3206_3207insG in *PIK3CA* which results in gain of function by provoking the PI3K/AKT/mTOR pathway. According to the previous reports, over 80% of somatic, activating *PIK3CA* mutations in solid cancers and benign overgrowth disorders cluster at three residues, two glutamic acids (E) residues at codons 542 and 545, and a histidine (H) residue at codon 1047. Three hotspot mutations E542K, E545K, and H1047R underlie over 2/3 of patients with *PIK3CA-*associated cancers and PROS. To the best of our knowledge, this is the first report of a frameshift mutation in *PIK3CA* causing CLOVES syndrome. Unlike a great mount of frameshift mutations that usually producing premature loss-of-function truncated protein, there are multiple C-terminal frameshift somatic mutations annotated in the COSMIC database including various insertions affecting the stop codon. For instance, in our study, the mutation is located in the stop codon of *PIK3CA*, which is predicted to eliminate the canonical translation termination by generating a prolonged protein. qRT-PCR confirmed that the expression of *PIK3CA* mRNA from the patient’s skin lesion was comparable to the normal control, suggesting that the mutation escapes nonsense-mediated mRNA decay. Further functional studies showed that, in consistent with other reported PROS-associated high-frequent *PIK3CA* mutations, this frameshift mutation provoked AKT phosphorylation, resulting in elevated PI3K/AKT/mTOR activity.

According to a recent clinical trial, six individuals with Proteus syndrome were treated with oral Miransertib (ARQ092) and the appropriate dosage was evaluated by using a pharmacodynamic endpoint [23]. Proteus syndrome is caused by somatic activating mutations in *AKT1*, which leads to overactivation of PI3K/AKT/mTOR signaling pathway as PROS-associated *PIK3CA* mutations [24, 25]. ARQ092 was demonstrated to decrease the phosphorylation of AKT. Patients with Proteus syndrome were well tolerated at a dose of 5 mg/m^2^/day ARQ092, suggesting that this kind of pan AKT inhibitor might be an alternative effective approach to PROS. This concept was further strengthened by our study which showed that provoked AKT phosphorylation due to PIK3CA-X1069Wfs*4 could be effectively inhibited by ARQ092, even stronger than rapamycin. Of note, ARQ092 has now been granted “Fast Track” designation by the Food and Drug Administration (FDA) for clinical treatment of PROS as well as Proteus syndrome, which is the first Pan-AKT inhibitor approved for treating PROS and Proteus syndrome. Before this, inhibiting mTOR is the only therapeutic approach for inhibiting PI3K/AKT/mTOR signaling approved by FDA. Further investigations should be taken to study the efficacy and safety of molecule drugs for PROS and other overgrowth syndromes.

In summary, our results have expanded the mutation spectrum of CLOVES syndrome and suggested a potential alternative therapeutic drug ARQ092 for PROS.

## Conclusions

We report a case of CLOVES syndrome due to a frameshift mutation c.3206_3207insG in *PIK3CA*, which was confirmed to be gain-of-function by provoking PI3K/AKT/mTOR signaling pathway*.* The inhibitory effect of ARQ092 in elevated AKT phosphorylation caused by *PIK3CA* mutations provides a potential alternative therapeutic drug for PROS.

## Data Availability

All data used during the study appear in the submitted article.

## Abbreviations

PI3Ks: Phosphatidylinositol 3‑kinases
RTK: Receptor tyrosine kinase
GPCR: G‑protein coupled receptor (GPCR)
PROS: Overgrowth disorders termed PIK3CA-related overgrowth spectrum
mTOR: Mammalian target of rapamycin
FDA: Food and Drug Administratio
CST: Cell Signaling Technology
